# Local Immune Responses in Children and Adults with Allergic and Nonallergic Rhinitis

**DOI:** 10.1371/journal.pone.0156979

**Published:** 2016-06-09

**Authors:** Kyung Suk Lee, Jinho Yu, Dahee Shim, Hana Choi, Man-Young Jang, Kyung Rae Kim, Jae-Hoon Choi, Seok Hyun Cho

**Affiliations:** 1 Department of Pediatrics, CHA Bundang Medical Center, CHA University School of Medicine, Seongnam-si, Gyeonggi-do, Korea; 2 Department of Pediatrics, Childhood Asthma Atopy Center, Asan Medical Center, University of Ulsan College of Medicine, Seoul, Korea; 3 Department of Life Science, College of Natural Sciences, Research Institute for Natural Sciences, Seoul, Korea; 4 Department of Otorhinolaryngology-Head and Neck Surgery, College of Medicine, Hanyang University, Seoul, Korea; Beijing Institiute of Otolaryngology, CHINA

## Abstract

**Background:**

Allergic rhinitis (AR) is the most common allergic disease but little is known about the difference of local immune responses in children and adults with AR.

**Objective:**

To compare local immune responses between children and adults with AR and nonallergic rhinitis (NAR), and to investigate whether the association of local and systemic immune responses is different between the two age groups.

**Methods:**

Fifty-one patients with chronic rhinitis were enrolled and grouped into children (N = 27, mean age 7.2 years) and adults (N = 24, mean age 29.9 years). Diagnosis of AR was based on symptoms, skin prick tests and serum specific IgEs. Nasal lavage (NAL) fluids were collected from all subjects and used to measure the levels of total IgE, specific IgEs to house dust mites (Dp and Df), and cytokines (TNF-α, IL-4, IL-10, IL-17A and IFN-γ). Flow cytometry was used to measure inflammatory cell types in NAL fluids.

**Results:**

AR had significantly increased local levels of total IgE and specific IgEs to Dp and Df compared with NAR in both age groups (*P* < 0.05). Nasal eosinophils % (*P* = 0.01) was significantly increased only in children with AR. Local-systemic correlations of total IgE (*r* = 0.662, *P* = 0.000) and eosinophil % (*r* = 0.461, *P* = 0.015) between the peripheral blood and NAL fluids were found only in children. Moreover, children had correlations between total IgE and eosinophil % in the peripheral blood (*r* = 0.629, *P* = 0.001) and in NAL fluids (*r* = 0.373, *P* = 0.061).

**Conclusion:**

Elevated local IgE is a common feature of AR in children and adults. Local measures in NAR showed naïve state of immune response which disagree with the hypothesis of local allergic rhinitis. Children showed intense local inflammation and close local-systemic interactions compared to adults supporting pediatric AR as a distinct feature.

## Introduction

Allergic rhinitis (AR) is the most common allergic disease with a high economic burden [1]. AR has been reported as to affect 36%-40% of children and 10%-30% of adults [2–5]. The prevalence of AR has progressively increased in Westernized countries over the past three decades.

AR can be diagnosed by history and clinical symptoms, and through the demonstration of specific IgE to common inhalant allergens by skin prick tests (SPTs) and in vitro assays [6]. When the results of these tests are negative, rhinitis is classified into nonallergic rhinitis (NAR), including a heterogeneous group of infectious, occupational and vasomotor rhinitis [7]. Recently, local allergic rhinitis (LAR) has been emerging as a new endotype of rhinitis, characterized by positive responses to nasal provocation tests and/or local synthesis of specific IgE in the absence of systemic atopy [1, 8]. Thus, the current scheme for differential diagnosis of rhinitis, which is dependent on IgE-mediated systemic immune response, does not seem to be sufficient. In addition, roles of the local immune response and the association between local and systemic counterparts need to be elucidated in patients with AR and NAR.

Nasal lavage (NAL) is a non-invasive procedure to collect nasal secretions, which provides information on local nasal immunity in patients with rhinitis. House dust mite-specific IgE was observed in the nasal secretions of adults with NAR as well as those with AR [9]. The finding of rye grass pollen-specific IgG, IgA, and IgE in the nasal secretions and sera of patients with AR suggests that the nasal mucosa may be the source of allergen-specific IgE in peripheral blood [10]. Although a few studies have measured nasal cytokines in children with AR [11, 12], little is known about local immunity in children with AR. Moreover, there is no information available on local immunity changing from children to adults with AR.

AR can develop during early childhood and the prevalence decreases with age [13, 14]. Allergic symptoms and skin test reactivity become milder over time [15]. Thus, nasal inflammation in AR is not simply caused by IgE-mediated eosinophilic inflammation, but rather it is a dynamic and chronic process involving interactions with environmental factors such as allergens, microbes and air pollutants [16, 17]. Moreover, local immune responses can be continually balancing over the two axes of pro-inflammatory and regulatory directions.

The aims of this study were to compare local immune responses between children and adults with AR and NAR, and to investigate whether the association of local immune response with systemic parameters is different with aging process.

## Materials and Methods

### Patients

We enrolled 51 consecutive patients (27 children and 24 adults) with AR or NAR between October 2013 and February 2014. Exclusion criteria included chronic rhinosinusitis (with or without nasal polyposis), fungal sinusitis, asthma, aspirin intolerance, history of nasal surgery, and paranasal sinus tumors. Adults (male 83.3%, age range 18–60, mean age 29.9 years) were comorbid with septal deviation and children (male 62.9%, age range 3–13, mean age 7.2 years) were comorbid with adenotonsillar hypertrophy. None of the subjects used antibiotics or nasal corticosteroids within 4 weeks.

All patients had rhinitis symptoms, including nasal obstruction, rhinorrhea and sneezing, and underwent assays for serum total IgE and complete blood count (CBC). SPTs were performed to determine their sensitivity to commonly inhaled allergens and the reaction was regarded as positive if the mean wheal diameter was 3 mm or greater [18]. The diagnosis of AR and NAR was defined according to the recently published criteria [19]. Nasal endoscopy and computed tomography were performed for differential diagnosis in adults.

Two Consent forms were prepared for children aged **≥** 13 years and children aged 7–12 years to understand objects, benefits, and harms of this study. And we obtained informed consents from both children and their parents. We used questionnaire reporting their nasal symptoms (itching, sneezing, rhinorrhea, and obstruction) with four-point scale (0 = no symptom; 1 = mild; 2 = moderate; 3 = severe) [20]. The total nasal symptom score (TNSS) was calculated as the sum of the scores in each domain. Quality of life (QOL) was measured on a 10-point visual analog scale (VAS). The study was approved by the Institutional Review Board of Hanyang University Medical Center (HYI 12–012).

### Tissue sampling

Nasal lavage was performed as described previously [21]. Briefly, after induction of general anesthesia with head-down position, each nostril was flushed with 5 ml of isotonic saline. The fluid was aspirated with a suction collector under the nasal endoscopy and immediately transferred to the laboratory bench. Crust and debris were removed with a cell strainer (100 μm, SPL, Pocheon, Gyeonggi-do, South Korea) and the fluids were immediately centrifuged at 6,000 rpm for 5 min. The supernatants were decanted and stored at -80°C. The cell pellets were resuspended in 1 ml of Dulbecco’s phosphate buffered saline (PBS) containing 2% fetal bovine serum (FBS) for flow cytometry.

### Flow cytometry

**Fig 1:**
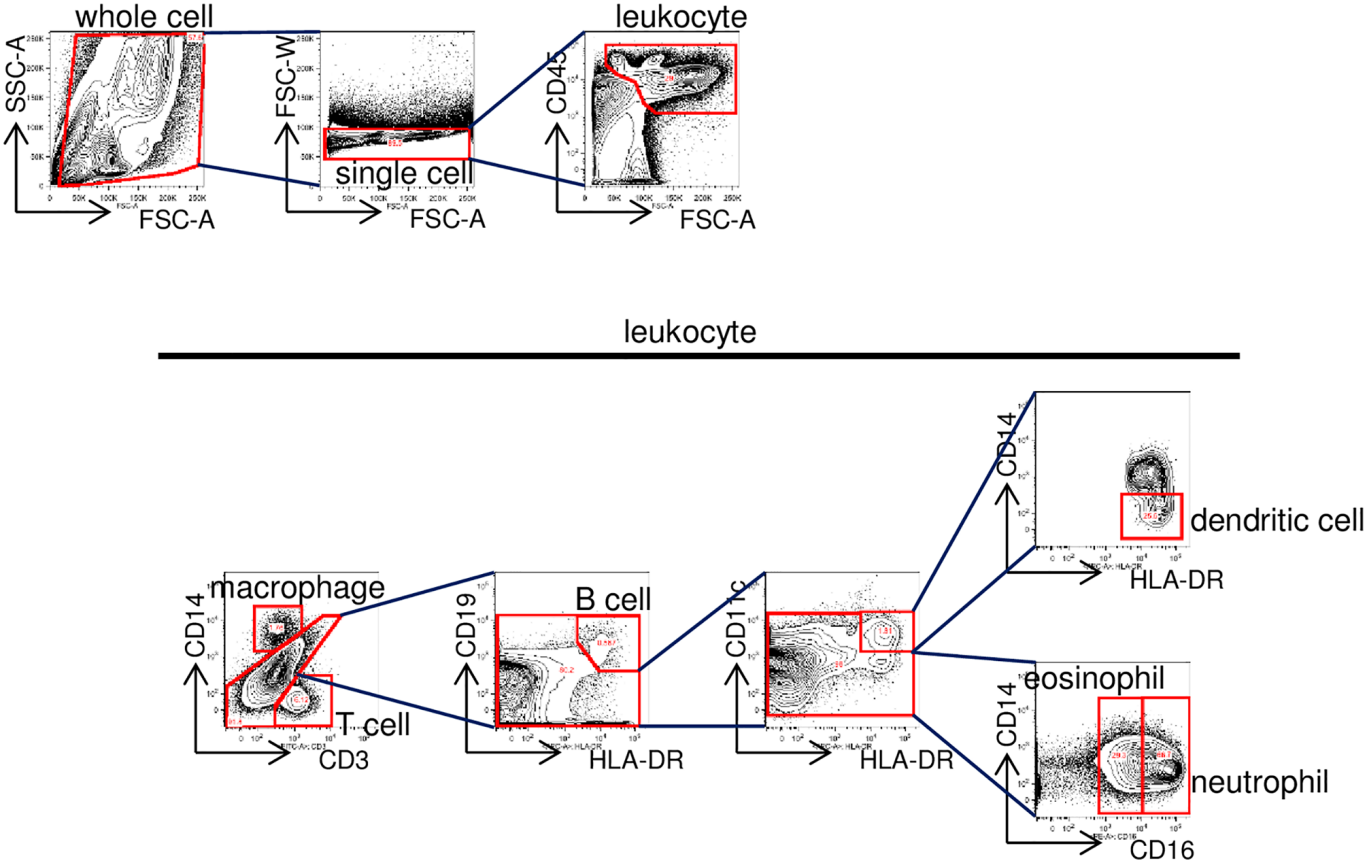
Gating strategy. Cell populations were incubated with antibodies against CD45, CD3, CD14, CD19, HLA-DR, CD11c, and CD16. The expression of surface markers on CD45^+^ leukocytes was analyzed by flow cytometry: CD3^-^ CD14^+^ macrophages, CD3^+^ CD14^-^ T cells, CD3^-^ CD14^-^ CD19^+^ HLA-DR^+^ B cells, CD3^-^ CD19^-^ CD11c^+^ HLA-DR^+^ CD14^-^ dendritic cells, CD3^-^ CD14^-^ CD19^-^ CD11c^-^ CD16^high^ neutrophils, and CD3^-^ CD14^-^ CD19^-^ CD11c^-^ CD16^intermediate^ eosinophils.

Non-specific antibody binding was blocked by incubating cells with TruStain FcX (eBioscience, San Diego, CA, USA) on ice for 15 minutes. The cells were subsequently incubated with monoclonal antibodies (Table 1) on ice for 30 minutes. The monoclonal antibodies included anti-human CD45 (HI30, BioLegend, San Diego, CA, USA) for whole leukocytes, anti-human CD3 (HIT3a, BD Pharmingen, San Jose, CA, USA) for T cells, anti-human CD14 (HCD14, M5E2, BioLegend, San Diego, CA, USA) for macrophages, anti-human CD19 (HIB19, BioLegend, San Diego, CA, USA) for B cells, anti-human CD11c (3.9, BioLegend, San Diego, CA, USA) for dendritic cells, anti-human CD16 (3G8, BioLegend, San Diego, CA, USA) for granulocytes including neutrophils and eosinophils, and anti-human HLA-DR (L243, BioLegend, LN3, eBioscience, San Diego, CA, USA) for dendritic cells and B cells. Flow cytometry was performed using a FACS Canto II (BD Biosciences, San Jose, CA, USA) and data were analyzed using Flow Jo ver. 9.3.2 (Treestar, Inc., San Carlos, CA, USA) (Fig 1).

**Table 1:** Antibody panel for leukocyte surface proteins associated with nasal lavage cells.

Target molecule	Host	Clone	Company
HLA-DR	mouse	L243	BioLegend
HLA-DR	mouse	LN3	eBioscience
CD3	mouse	HIT3a	BD Pharmingen
CD11c	mouse	3.9	BioLegend
CD14	mouse	HCD14	BioLegend
CD14	mouse	M5E2	BioLegend
CD16	mouse	3G8	BioLegend
CD19	mouse	HIB19	BioLegend
CD45	mouse	HI30	BioLegend

### CAP measurement of immunoglobulins, superantigens, and ECP in the NAL fluids

To evaluate local antibody production, we measured total IgE and specific IgEs for house dust mites (*Dermatophagoides pteronyssinus*; Dp and *Dermatophagoides farinae*; Df) and superantigens (staphylococcus enterotoxins A; SEA and staphylococcus enterotoxins B; SEB) in NAL fluids. To monitor the allergic response, we also measured eosinophil cationic peptide (ECP) in NAL fluids. All analyses were performed using the ImmunoCAP system (Phadia, Uppsala, Sweden). IgE concentrations were expressed as kU/L and ECP as μg/L [22, 23].

### Enzyme-linked immunosorbent assays (ELISA)

The concentrations of interleukin-4 (IL-4), IL-10, IL-17A, interferon-γ (IFN-γ) (R&D Systems, Minneapolis, MN, USA), and tumor necrosis factor-α (TNF-α, BD Biosciences, San Jose, CA, USA) were measured in NAL fluids using commercially available ELISA kits, according to the manufacturers' protocols.

### Statistics

Because of the relatively small sample size and the non-normal distribution, continuous variables are presented as medians with/without an interquartile range instead of as means with/without standard deviations. Statistical significance (*P* < .05) was analyzed using the Mann-Whitney *U* test. The relationships between systemic and local allergic parameters were examined using Spearman correlation analysis. Receiver-operating characteristic (ROC) curve analysis was used to determine the AR cutoff in adults and children with optimal sensitivity and specificity. All statistical analyses were performed using Graph-Pad Prism ver. 5.0 (Graph-Pad Software Inc., San Diego, CA, USA) and IBM SPSS ver. 21.0 (IBM Co., Armonk, NY, USA).

## Results

### Clinical characteristics of subjects with AR and NAR

The clinical characteristics of the study subjects are summarized in Table 2. The AR group was younger in adults (*P* = 0.011) but older in children (*P* = 0.043) than their respective NAR group. Sex (%), TNSS, and QOL were not significantly different between AR and NAR in either age group. House dust mites (Dp and Df) were the most common allergen in children (100%) and adults (73.3%) with AR. There was no correlation between TNSS and nasal IgE levels regardless of rhinitis types and age groups (not shown). The volumes of NAL fluids recovered after washing the nasal cavity with 10 mL of saline were not significantly different among the groups.

**Table 2:** Epidemiologic and clinical characteristics of the study subjects.

Characteristics	Children		Adults	
	NAR	AR	NAR	AR
N	12	15	9	15
Sex (M:F)	10:2	7:8	7:2	13:2
Age (yr)	5.0 (4.0–7.8)	8.0 (6.0–11.0)	36.0 (26.0–49.5)	23 (19–33.0)
TNSS	5.5 (4.0–7.8)	6.0 (4.0–8.0)	7.0 (3.0–7.0)	7.0 (5.0–9.0)
QOL	5.0 (4.3–5.0)	5.0 (4.0–6.0)	4.0 (2.0–6.0)	4.0 (2.5–5.0)
Skin prick test				
Dp	0%	86.7%	0%	60%
Df	0%	93.3%	0%	73.3%
pollens	0%	6.7%	0%	33.3%
cat or dog	0%	20%	0%	26.7%

Continuous variables are presented as median with interquartile range. NAR: nonallergic rhinitis, AR: allergic rhinitis, TNSS: total nasal symptom score, QOL: quality of life, Dp: *Dermatophagoides pteronyssinus*, Df: *Dermatophagoides farinae*.

The levels of serum total IgE (*P* = 0.016) and blood eosinophil % (*P* = 0.001) were significantly increased in AR compared to NAR in children (Table 3). However in adults, they were not different between AR and NAR (Table 4).

**Table 3:** Systemic (blood) and local (nasal lavage fluid) immune parameters in children with NAR and AR.

	Parameters	Children NAR (n = 12)	Children AR (n = 15)	*P* value
Systemic immunity	Total IgE (IU/ml)	41.9 (19.4–193.3)	269.7 (96.9–559.5)	0.016*
	Eosinophils (%)	1.8 (1.1–2.3)	5.6 (3.5–8.0)	0.001**
Local cellular immunity	WBC count (/mm^3^)	92,558 (36,320–156,962)	47,471 (26,493–144,849)	0.495
	Macrophages (%)	1.6 (0.8–3.1)	0.6 (0.3–0.9)	0.005**
	Dendritic cells (%)	2.5 (0.4–3.7)	1.6 (0.7–2.6)	0.884
	T cells (%)	3.4 (2.6–7.4)	3.3 (2.1–7.0)	0.733
	B cells (%)	17.0 (7.1–35.3)	13.5 (9.5–20.3)	0.696
	Neutrophils (%)	37.8 (23.8–51.5)	38.4 (25.0–57.0)	0.845
	Eosinophils (%)	9.5 (4.8–13.0)	15.0 (11.4–23.5)	0.010*
Local humoral immunity	Total IgE (kU/L)	4.00 (3.25–4.20)	8.14 (4.88–16.78)	0.001**
	Dp-IgE (kU/L)	0.10 (0.07–0.13)	0.86 (0.29–4.58)	0.001**
	Df-IgE (kU/L)	0.12 (0.08–0.12)	2.41 (0.79–4.44)	0.001**
	SEA-IgE (kU/L)	0.08 (0.07–0.09)	0.10 (0.07–0.10)	0.209
	SEB-IgE (kU/L)	0.07 (0.05–0.08)	0.08 (0.07–0.09)	0.159
	ECP (μg/L)	31.0 (15.1–82.6)	59.65 (27.90–151.50)	0.149
	TNF-α (pg/ml)	2.07 (0.00–22.94)	0.24 (0.00–4.97)	0.465
	IL-4 (pg/ml)	0.08 (0.00–2.53)	0.72 (0.00–3.08)	0.550
	IL-10 (pg/ml)	14.41 (3.90–100.95)	3.51 (0.00–17.04)	0.055
	IL-17A (pg/ml)	0.00 (0.00–0.75)	0.00 (0.00–2.43)	0.947
	IFN-γ (pg/ml)	5.89 (0.00–50.71)	19.29 (2.77–28.57)	0.422

Continuous variables are presented as median with interquartile range. NAR: nonallergic rhinitis, AR: allergic rhinitis, Dp: *Dermatophagoides pteronyssinus*, Df: *Dermatophagoides farina*, SEA: *Staphylococcus* enterotoxin A, SEB: *Staphylococcus* enterotoxin B, ECP: eosinophil cationic protein, TNF-α: tumor necrosis factor-alpha, IL: interleukin, IFN-γ: interferon-gamma.

*P < 0.05

**P < 0.01

**Table 4:** Systemic (blood) and local (nasal lavage fluid) immune parameters in adults with NAR and AR.

	Parameters	Adults NAR (n = 9)	Adults AR (n = 15)	*P* value
Systemic immunity	Total IgE (IU/ml)	53.0 (34.8–153.7)	286.20 (50.9–845.6)	0.121
	Eosinophils (%)	2.0 (1.6–2.9)	3.2 (1.1–4.1)	0.455
Local cellular immunity	WBC count (/mm^3^)	10,733 (3,960–98,313)	14,947 (6,155–27,781)	0.655
	Macrophages (%)	1.0 (0.4–8.2)	0.50 (0.2–1.5)	0.161
	Dendritic cells (%)	1.5 (0.7–2.8)	0.8 (0.4–1.1)	0.060
	T cells (%)	15.4 (4.6–34.9)	22.3 (5.4–37.6)	0.788
	B cells (%)	5.2 (4.2–14.1)	5.2 (1.2–9.6)	0.633
	Neutrophils (%)	60.0 (24.1–71.0)	40.6 (27.4–70.3)	0.698
	Eosinophils (%)	4.7 (2.2–12.8)	3.9 (1.3–17.6)	0.698
Local humoral immunity	Total IgE (kU/L)	4.36 (3.09–4.71)	6.95 (5.30–11.60)	0.019*
	Dp-IgE (kU/L)	0.14 (0.07–0.14)	0.24 (0.18–1.09)	0.006**
	Df-IgE (kU/L)	0.12 (0.08–0.13)	0.30 (0.17–1.58)	0.003**
	SEA-IgE (kU/L)	0.07 (0.06–0.09)	0.09 (0.07–0.10)	0.184
	SEB-IgE (kU/L)	0.07 (0.06–0.09)	0.08 (0.07–0.10)	0.117
	ECP (μg/L)	17.1 (5.2–42.4)	34.0 (15.6–73.4)	0.101
	TNF-α (pg/ml)	5.92 (0.00–19.09)	1.10 (0.00–4.05)	0.188
	IL-4 (pg/ml)	0.72 (0.00–4.06)	1.28 (0.00–6.28)	0.692
	IL-10 (pg/ml)	0.00 (0.00–33.13)	0.00 (0.00–0.00)	0.071
	IL-17A (pg/ml)	0.00 (0.00–0.00)	0.00 (0.00–0.00)	0.795
	IFN-γ (pg/ml)	15.71 (1.25–63.57)	4.64 (0–11.79)	0.176

Continuous variables are presented as median with interquartile range. NAR: nonallergic rhinitis, AR: allergic rhinitis, Dp: *Dermatophagoides pteronyssinus*, Df: *Dermatophagoides farinae*, SEA: *Staphylococcus* enterotoxin A, SEB: *Staphylococcus* enterotoxin B, ECP: eosinophil cationic protein, TNF-α: tumor necrosis factor-alpha, IL: interleukin, IFN-γ: interferon-gamma.

*P < 0.05

**P < 0.01

### Local immune response in the nasal airway of children with AR

In children, flow cytometry of the NAL fluids showed a significant increase in eosinophils (*P* = 0.01) and a decrease in macrophages (*P* = 0.005) in AR compared with NAR (Table 3). CAP analysis of the NAL fluids showed significantly increased levels of total IgE (*P* = 0.01), Dp-specific IgE (*P* = 0.001) and Df-specific IgE (*P* = 0.001) in AR compared with NAR. Macrophages (%) had negative correlations with total IgE (*r* = -0.558, *P* = 0.003), Dp-IgE (*r* = -0.446, *P* = 0.023), and Df-IgE (*r* = -0.445, *P* = 0.023) in NAL fluids. However, there was no difference in the levels of specific IgE to superantigens (SEA and SEB), ECP, and other T cell cytokines (including IL-10) in NAL fluids between the subgroups in children.

### Local immune response in the nasal airway of adults with AR

In adults, CAP analysis of the NAL fluids showed significantly increased levels of total IgE (*P* = 0.019), Dp-specific IgE (*P* = 0.006) and Df-specific IgE (*P* = 0.003) in AR compared with NAR (Table 4). However, no inflammatory cells including macrophages, dendritic cells, and eosinophils were different in the NAL fluids between AR and NAR. In addition, there was no difference in the levels of specific IgE to superantigens (SEA and SEB), ECP, and other T cell cytokines (including IL-10) in NAL fluids between the subgroups in adults.

### Comparison of local and systemic immune parameters between children and adults

**Fig 2:**
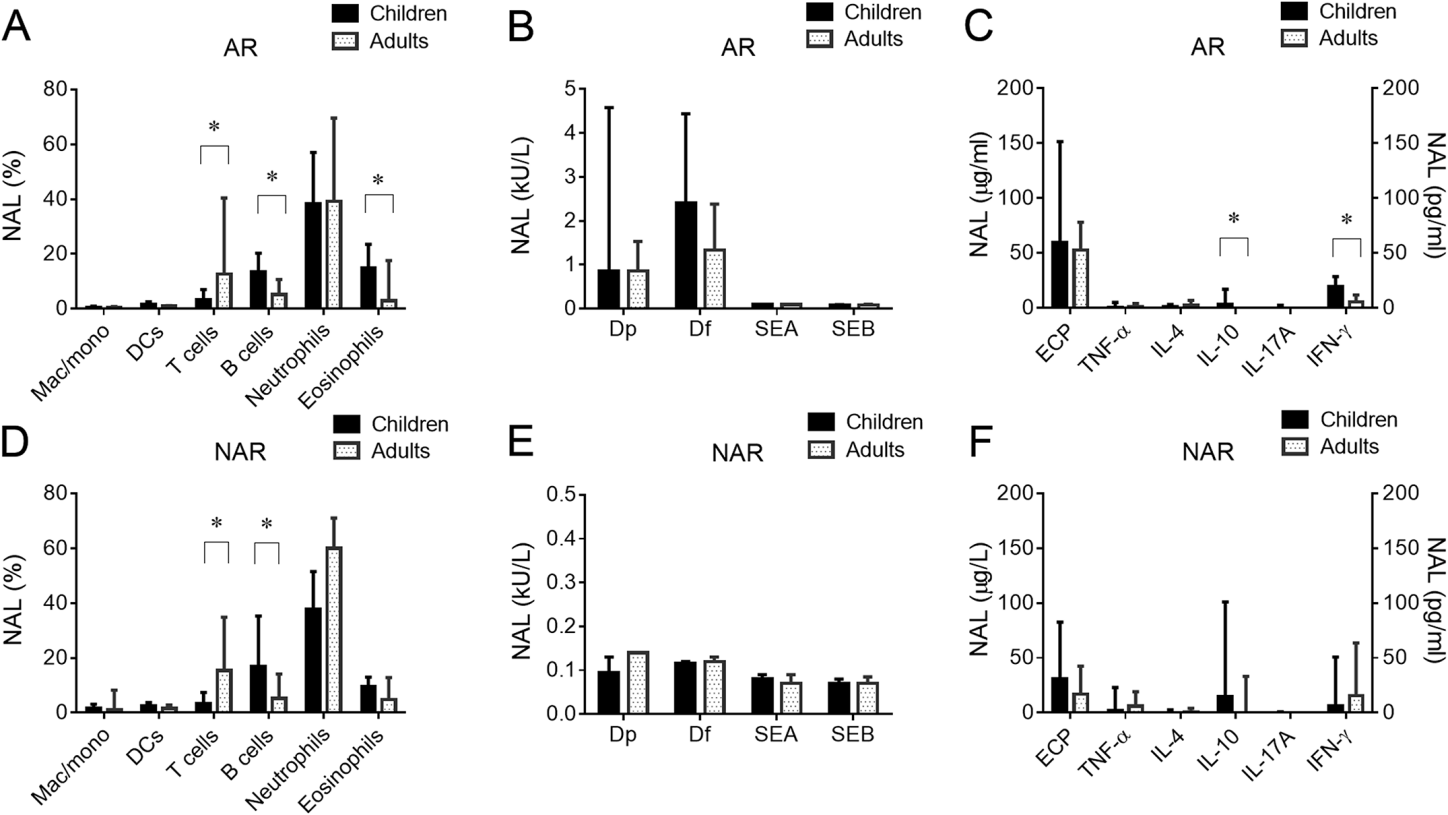
Local immune parameters in children and adults with allergic rhinitis (AR, A-C) and nonallergic rhinitis (NAR, D-F). (A, D), flow cytometry; (B, E), CAP immunoassay; (C, F), ELISA. *P < 0.05, **P < 0.01.

To compare the local and systemic immune responses between children and adults, we performed subgroup analyses of subjects with AR sensitized to only house dust mites. Blood eosinophil % (*P* = 0.02) were significantly higher in children than in adults, but the levels of serum total IgE were not different between the two groups (*P* = 0.827). In NAL fluids, eosinophil % (*P* = 0.036) were significantly increased in children compared to adults (Fig 2A). However, the levels of total IgE and specific IgEs to house dust mites were not different between the two groups (Fig 2B). Nasal concentrations of IL-10 (*P* = 0.021) and IFN-γ (*P* = 0.038) were significantly increased in children compared to adults (Fig 2C). However, the local levels of specific IgE to super-antigens, ECP, and other T-cell cytokines were not significantly different between the two groups (Fig 2B and 2C).

Regardless of atopic status, flow cytometry of the NAL fluids showed a significant increase in B cells and a decrease in T cells in children compared to adults (Fig 2A and 2D). In the NAR groups, the local measurements of other parameters did not show any differences between children and adults (Fig 2D–2F).

### Association of local and systemic immune responses in children and adults

**Fig 3:**
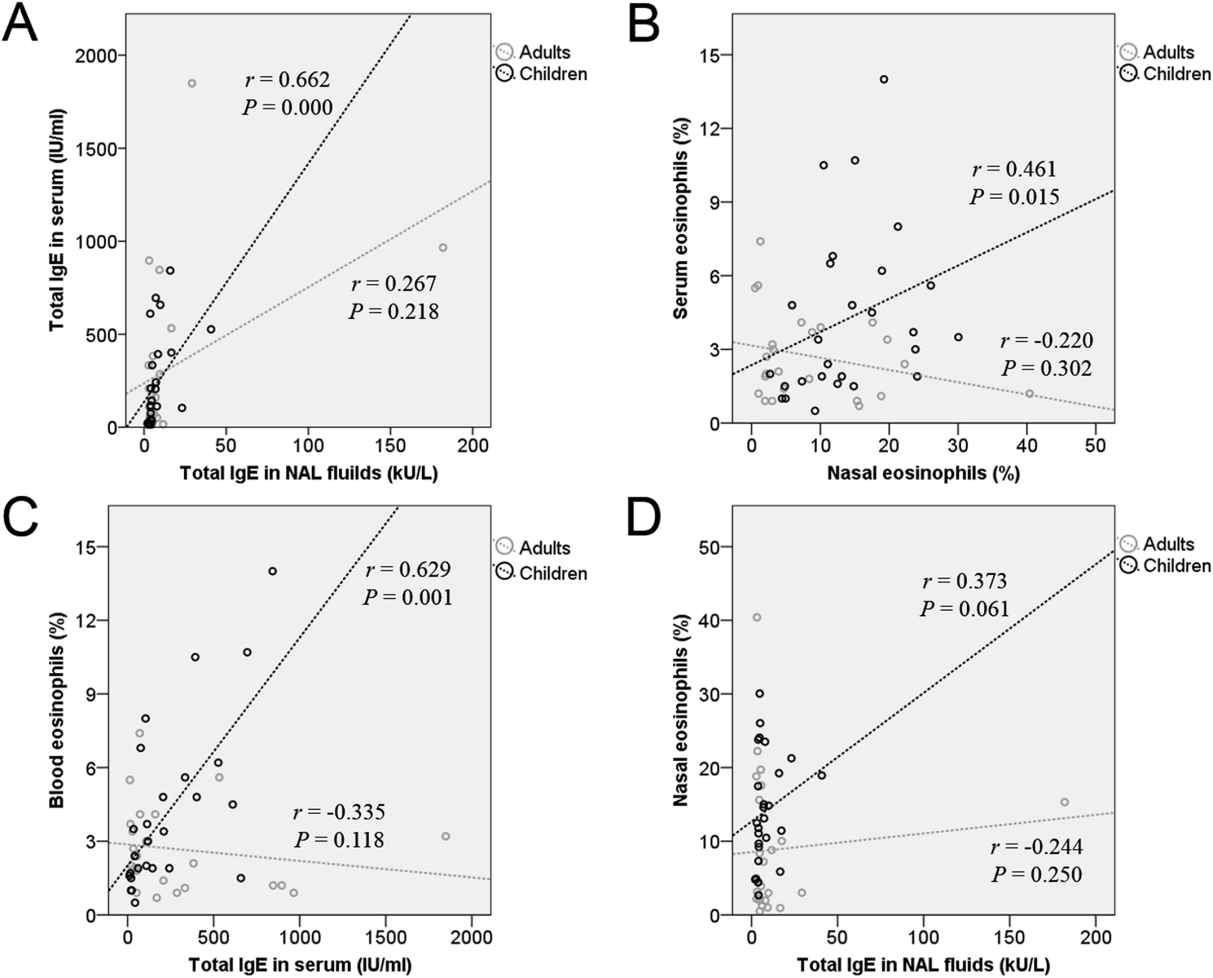
Correlation analysis of local-systemic interactions in children and adults. (A) Scatter plots and regression lines show interactions of (A) total IgE between NAL fluids and serum and (B) eosinophilia (%) between NAL fluid and peripheral blood. IgE-mediated eosinophilic inflammation is shown in the peripheral blood (C) and NAL fluids (D).

We found significant local-systemic correlations of total IgE (*r* = 0.662, *P* = 0.001, Fig 3A) and eosinophil % (*r* = 0.461, *P* = 0.015, Fig 3B) between the NAL fluids and peripheral blood in children. However, these correlations were not found in adults (Fig 3A and 3B). In children, the correlation between total IgE and eosinophil % was significant in the peripheral blood (*r* = 0.629, *P* = 0.001, Fig 3C) but not in the NAL fluids (*r* = 0.373, *P* = 0.061, Fig 3D). And these findings were also not found in adults (Fig 3C and 3D).

### ROC curves of local immune parameters for AR in children and adults

ROC curves of the local immune parameters were constructed to evaluate their ability to diagnose AR in children and adults based on their cut-off values, sensitivity, and specificity (Table 5). The areas under the curves (AUC) for the diagnosis of AR were significant for total IgE (*P* = 0.001 and *P* = 0.004, respectively), and specific IgEs to Dp (*P* = 0.001 and *P* = 0.007, respectively) and Df (*P* = 0.001 and *P* = 0.005, respectively) measured in NAL fluids from children and adults. However, nasal eosinophils % (cut-off = 13.9%, AUC = 0.821, *P* = 0.007) was significant for the diagnosis of AR only in children.

**Table 5:** Cut-off value and areas under the receiver operating characteristics for local immune markers in children and adults with AR.

Immune markers	Cut-off	Sensitivity	Specificity	AUC	*P* value
Children AR					
Total IgE (kU/L)	4.65	0.846	0.917	0.891	0.001**
Dp-IgE (kU/L)	0.18	0.846	1.000	0.939	0.001**
Df-IgE (kU/L)	0.28	0.846	1.000	0.949	0.001**
Eosinophils (%)	13.86	0.769	0.833	0.821	0.007**
Adults AR					
Total IgE (kU/L)	4.69	0.867	0.875	0.875	0.004**
Dp-IgE (kU/L)	0.16	0.800	1.000	0.846	0.007**
Df-IgE (kU/L)	0.14	0.867	0.875	0.867	0.005**
Eosinophils (%)	3.53	0.533	0.500	0.467	0.796

AR: allergic rhinitis, Dp: *Dermatophagoides pteronyssinus*, Df: *Dermatophagoides farinae*.

*P < 0.05

**P < 0.01

## Discussion

This study analyzed cellular and humoral immune responses in NAL fluids from children and adults with AR and NAR, and their local-systemic associations. Additionally, we investigated whether children with AR have a different immunologic phenotype from adults with AR. Our findings can be summarized as follows: (1) Increased production of total and allergen-specific IgEs was found in the nasal airways of AR compared with NAR in both age groups. (2) In AR, children had higher levels of eosinophilic inflammation in both peripheral blood and the nasal airway than adults. (3) Local-systemic correlations of total IgE and eosinophil % were significant only in children.

AR is a hypersensitivity disease mediated by specific IgE when exposed to inhalant allergens [6]. IgE is capable to crosslink the high and low affinity receptor (FcεRI) of effector cells (mast cells and basophils), and starts allergic cascades resulting rhinitis symptoms such as sneezing, rhinorrhea and nasal obstruction [6, 24]. Although systemic and skin IgE are widely used as in vivo and in vitro tests, it has been not fully investigated about age-related roles of local IgE and its systemic interactions.

Increased levels of total IgE and specific IgEs to house dust mites were found in the NAL fluids from children and adults with AR compared to levels measured in their respective NAR groups. Our findings support the fact that local IgE is a key molecule that drives nasal eosinophilic inflammation in patients with AR [25, 26]. However, only children with AR showed increased local eosinophilia, and the significant correlation between total IgE and eosinophils in NAL fluids. Thus, when compared to adults, children with AR seem to have severe atopic intensity and strong IgE-mediated eosinophilic inflammation.

Local and systemic levels of total IgE and eosinophils showed significant associations in children but not in adults. Local eosinophilic inflammation may precede systemic eosinophilic inflammation, as local IgE is the source of serum IgE. Consistent with previous findings [27, 28], the hypothesis of “local-systemic interactions” was also observed in our study. However, we found discrepancies of local-systemic interactions between children and adults. Previous studies have reported that children had higher levels of serum total IgE and eosinophils (%) compared to adults which is consistent to our results [29–31]. Innate and adaptive immune response can be altered or impaired with increased age, and therefore, our results provided an evidence supporting the phenomenon of “immunosenescence” in allergic rhinitis which can explain the loss of local-systemic interactions in adults [31].

Specific IgEs for inhalant allergens (e.g. house dust mite) can be locally produced and spill over into systemic circulation [28]. Flow cytometry showed plenty of B lymphocytes in NAL fluids, which may be the source of local IgEs. Because we measured not the membrane-bound IgE but the secreted form of local IgE, we could not demonstrate their direct roles in the pathogenesis of AR which needs further investigation.

LAR is characterized by the presence of nasal Th2 inflammation with local production of specific IgEs and/or a positive response to a nasal allergen provocation test without evidence of systemic atopy [8]. Previous studies, mostly performed in Western populations, have reported the prevalence of LAR to be 25.7% in chronic rhinitis [32] and 47%-62.5% in NAR [33–35]. In this study, we found that local IgEs (total and allergen-specific) were elevated only in AR of children and adults. In other word, all patients with NAR had low level of local IgE and there was no case correspondent to LAR. Therefore, we confirmed roles of local IgEs in AR but could not support the concept of local allergic rhinitis in NAR. This discrepant result is very important issue which needs to be re-evaluated by other researchers in Western and Asian countries.

Recent studies have shown that innate immune cells, like macrophages and dendritic cells, can induce Th2 responses. Macrophages can affect the production of IL-4 or immunoglobulin and the class switching of lymphocytes in the initiation of mouse AR [30]. In our study, flow cytometry showed that decreased nasal macrophages in children with AR compared to NAR. Moreover, macrophages had negative associations with nasal immunoglobulins (total and specific). Therefore, macrophages may be actively involved in the regulation of allergic responses in children [36].

Enhanced Th2 cytokine responses and lower levels of IFN-γ from Th1 cells have been reported in NAL fluid from patients with AR [37–41]. However, our study showed that the levels of nasal Th-related cytokines (Th1, Th2, and Th17) did not differ between patients with AR and NAR regardless of age group. Local level of IL-10 measured in AR showed declining tendencies compared to NAR in both children and adults. Moreover, children with AR had increased local levels of IL-10 and IFN-γ compared to adults with AR. Therefore, we concluded that active pro-Th2 and regulatory responses may occur simultaneously in the nasal airway in children with AR. In adults, these two axes of immune response wane with aging process.

In conclusion, elevated local IgE is a common feature of AR in children and adults. Local measures in NAR showed naïve state in immune perspectives having very low levels of all cytokines and immunoglobulins which disagree with the hypothesis of “local allergic rhinitis”. In addition, children showed intense local inflammation and close local-systemic interaction compared to adults which support the phenomenon of “immunosenescence” and also suggest pediatric AR as a distinct feature.
